# A clinical utility assessment of the automatic measurement method of the quality of Meibomian glands

**DOI:** 10.1186/s12938-017-0373-4

**Published:** 2017-06-24

**Authors:** Robert Koprowski, Lei Tian, Paweł Olczyk

**Affiliations:** 1 Department of Biomedical Computer Systems, Faculty of Computer Science and Materials Science, Institute of Computer Science, University of Silesia, ul. Będzińska 39, 41-200 Sosnowiec, Poland; 2 Beijing Institute of Ophthalmology, Beijing Tongren Eye Center, Beijing Tongren Hospital, Capital Medical University, Beijing, 100730 China; 3 Beijing Ophthalmology & Visual Sciences Key Laboratory, Beijing, 100730 China; 4 Department of Community Pharmacy, School of Pharmacy with the Division of Laboratory Medicine in Sosnowiec, Medical University of Silesia in Katowice, Katowice, Poland

**Keywords:** Biomedical, Image processing, Meibomian gland dysfuction, Dry eye, Automatic analysis

## Abstract

**Background:**

Meibomian gland dysfunction (MGD) is one of the most common diseases observed in clinics and is the leading cause of evaporative dry eye. Today, diagnostics of MGD is not fully automatic yet and is based on a qualitative assessment made by an ophthalmologist. Therefore, an automatic analysis method was developed to assess MGD quantiatively.

**Materials:**

The analysis made use of 228 images of 57 patients recorded by OCULUS Keratograph^®^ 5 M with a resolution of 1024 × 1360 pixels concern 30 eyes of healthy individuals (14 women and 16 men) and 27 eyes of sick patients (10 women and 17 men). The diagnosis of dry eye was made according to the consensus of DED in China (2013).

**Methods:**

The presented method of analysis is a new, developed method enabling an automatic, reproducible and quantitative assessment of Meibomian glands. The analysis relates to employing the methods of analysis and image processing. The analysis was conducted in the Matlab environment Version 7.11.0.584, R2010b, Java VM Version: Java 1.6.0_17-b04 with Sun Microsystems Inc. with toolboxes: Statistical, Signal Processing and Image Processing.

**Results:**

The presented, new method of analysis of Meibomian glands is fully automatic, does not require operator’s intervention, allows obtaining reproducible results and enables a quantitative assessment of Meibomian glands. Compared to the other known methods, particularly with the method described in literature it allows obtaining better sensitivity (98%) and specificity (100%) results by 2%.

## Background

Meibomian gland is the largest sebaceous glands in the human body. Meibomian glands synthesize and secrete a mixture of lipids, termed meibomian oil or meibum [1], which is delivered as a clear liquid via orifices located directly in front of the mucocutaneous junction. Meibomian gland diseases are subdivided into focal lesion and diffuse lesion. The former refers to hordeolum or chalazion and the latter is meibomian gland dysfuction (MGD). MGD is a chronic, diffuse of meibomian gland lesions, of which pathological basis is meibomian gland terminal duct obstruction and (or) qualitative or quantitative change of meibum secretion [2]. It can cause abnormal tear film, eye irritation and ocular surface inflammation. More seriously, MGD can cause the ocular surface damage of visual function. At present, the prevalence of MGD in association with dry eye varies greatly, ranging from 3.5% in the Salisbury Eye Evaluation Study [3] to 60.8% in the Shihpai Eye Study in Taiwan [4], 68.3% in the Beijing Eye Study in China [5], and 56.3% in the Singapore Malay Eye Study [6]. MGD was highly in Asian population and associated with various systemic and ocular conditions, such as postmenopausal women, pinguecula, high diastolic blood pressure, anterior blepharitis, corneal contact lens and Demodex mites [6].

As we all know, the tear film lipid layer (TFLL) forms the outermost layer of the film and is composed of both polar and nonpolar lipids. It has been accepted that the TFLL is an indispensable component of the tear film, providing a smooth optical surface for the cornea and retarding evaporation from the eye [7]. Although the precise aetiology of MGD remain to be undetermined, as reported, MGD is a chronic, diffuse abnormality of the Meibomian glands, commonly characterised by terminal duct obstruction and (or qualitative) quantitative changes in the glandular secretion. It may result in alteration of the tear film, symptoms of eye irritation, clinically apparent inflammation, and ocular surface disease [8]. In a recent review, five separate pathophysiological mechanisms of MGD resulting from tear film instability were reported, eyelid inflammation, conjunctival inflammation, corneal damage, microbiological changes and dry eye disease (DED) [9]. The underlying pathophysiological mechanisms of DED and MGD interact, resulting in a double vicious circle. This is consistent with the high incidence of advanced stage MGD.

Dry eye disease is a common condition that occurs when the eyes don’t make enough tears, or the tears evaporate too quickly. It is now generally accepted that there are two major classifications of DED: aqueous deficient and evaporative dry eye [10–12]. Rather, DED a term that describes a range of conditions with multiple possible etiologies and comorbidities. Additionally, MGD is one of the most common diseases observed in clinics and is the leading cause of evaporative dry eye [13]. MGD occurs when the Meibomian glands, located in the eyelids, do not sufficiently produce and release the oils needed to protect and maintain a healthy tear film. This causes the watery layer in the tear film to evaporate. Thus, the problem for many dry eye patients is not inadequate tear production, as thought for so many years, but a lack of oil production that ensures the protective integrity of the tear film is maintained on a daily basis. Given the increased recognition of the importance of MGD, a great amount of attention has been paid to diagnosis and therapies targeting this condition.

## Materials

### Subject recruitment

The analysis made use of 228 images of 57 patients recorded by OCULUS Keratograph^®^ 5 M with a resolution of *M* × *N*=1024 × 1360 pixels (where *M*—the number of rows, *N*—the number of columns). The obtained images *L*_*RGB*_(*m*,*n,k*) (where *m*—row, *n*—column, *k*—color) concern 30 eyes of healthy individuals (14 women and 16 men) and 27 eyes of sick patients (10 women and 17 men). The diagnosis of dry eye was made according to the consensus of DED in China (2013): (1) at least 1 of 6 symptoms: dryness, burning, sandiness, tiredness, discomfort, and blurred vision with fluorescein tear breakup time ≤5 s or a non-anesthesia Schirmer I test value ≤5 mm/5 min; (2) at least 1 of 6 symptoms: dryness, burning, sandiness, tiredness, discomfort, and blurred vision with 5 s< fluorescein tear breakup time ≤10 s or 5 mm/5 min< non-anesthesia Schirmer I test ≤10 mm/5 min, accompanied by corneal fluorescein staining score. The diagnostic criteria of MGD were as follows: (1) symptoms of ocular discomfort: itching or photophobia with limitations of activities; (2) clinical signs: at least 1 lid margin feature (plugging, vascularity), meibum quality: grade: ≥4, expressibility: ≥1. Potential subjects were excluded from the study if they had undergone previous corneal or ocular surgery, had any ocular pathology other than DED and DI, had systemic diseases known to affect the eye. Participants were instructed to remove soft contact lenses at least 2 weeks and rigid contact lenses at least 1 month before the examination. Data were collected from August 2015 to May 2016 in Beijing Tongren Hospital, Beijing, China. All participants signed an informed consent form in accordance with the tenets of the Declaration of Helsinki and this study was approved by the institutional review board of Beijing Tongren Hospital, Beijing, China. The analysis was conducted in the Matlab environment Version 7.11.0.584, R2010b, Java VM Version: Java 1.6.0_17-b04 with Sun Microsystems Inc. with toolboxes: Statistical, Signal Processing i Image Processing.

### Examinations

Each patient finished 3 parts of the clinical examination: (1) Ocular surface evaluation: the Ocular Surface Disease Index (OSDI) Survey and fluorescein staining. (2) tear function assessment: fluorescein tear breakup time (BUT), non-anesthetized Schirmer I test (S|T); (3) meibomian gland function assessment: meibum quality, meibomian gland expressibility; meibomian gland dropout.

### Ocular surface evaluation

#### Ocular surface disease index

Before the other clinical examinations, patients were required to complete the Ocular Surface Disease Index (OSDI) Survey. This questionnaire gives a range of 0 (no symptoms) to 100 (severe symptoms).

#### Corneal fluorescein staining

Corneal fluorescein staining was graded from 0 to 12, a sum of the scores of corneal four quadrants, which were scored individually as 0 (no staining), 1 (mild staining with a few scattered dots of stains), 2 (moderate staining between 1 and 3), and 3 (severe staining with confluent stains or corneal filaments) [13].

### Tear function assessment

#### Schirmer I test

The SIT was a useful assessment of aqueous tear production. The inferior conjunctival fornix was dried with a cotton stick. One minute later, a standard 5 × 40 mm Schirmer test strip was placed over the junction of the middle and outer third of inferior lid. The patients are instructed to keep their eyes closed during the test. The test lasted 5 min, and the amount wetting was recorded [14].

#### Fluorescein tear breakup time

BUT is the time interval between the last blink and the appearance of the first random dry spot on the corneal surface after the instillation of fluorescein. The average of three consecutive FBUT values was calculated [15].

### Meibomian gland function measurement

The following three parameters were the most commonly used methods to evaluate the morphological characteristics and function of Meibomian glands in clinical practice: abnormalities of lid margins, expression of meibum, and gland dropout degree visualized by meibography [16].

#### Lid margin

Lid margin abnormality was recorded according to the existence of the following four signs: lid margin irregular, vascular engorgement, glandular orifices obstruction, and anterior or posterior displacement of the mucocutaneous junction, the scored was from 0 to 4 [17].

#### Meibum expression

The quality of the expression of meibum was assessed semiquantitatively in 8 glands of the central third area of the lower eyelids: Grade 0 clear fluid, Grade 1 cloudy fluid, Grade 2 cloudy, particulate fluid, and Grade 3 inspissated, toothpaste-like fluid [18].

## Methods and results

The presented method of analysis is a new, developed method enabling an automatic, reproducible and quantitative assessment of Meibomian glands [18–24]. The analysis relates to employing the methods of analysis and image processing [25–35]. The new, proposed method of image analysis was divided into three stages:

- Image acquisition,
- Initial image processing,
- Major image processing.

Particular stages will be comprehensively described in subsequent subunits.

### Image acquisition

Image acquisition was conducted in Beijing Tongren Hospital in China [36]. Each patient was sitting still in front of Keratograph 5 M. The ophthalmologist was simultaneously performing the examination and recording the images everting the upper and lower lid of the left and right eye of the patient. In total, 228 images of left and right eye of both upper and lower lid were obtained from 57 patients. The recorded sequence of images EX0000*.zip was automatically decompressed to acquire the images of lower *lowerLid.BMP* and upper lid *upperLid.BMP*. The names of the files are fixed for each patient. In the next stage of image processing, the algorithm works independently of the lid (lower or upper) and eye (right or left). The image inserted into the workspace (of lower or upper lid) will be further described as *L*_*RGB*_ (*m*,*n,k*).

### Initial image processing

Initial image processing was linked to three stages: filtering with median filter, normalization and removing uneven illumination [37–39]. Filtration with median filter was performed with the use of *h*_*MED*_ mask of the size *M*_*MED*_ × *N*_*MED*_ = 3 × 3 pixels [40]. The output image *L*_*MED*_ (*m*,*n*,*i*) was further normalized i.e. the image after normalization *L*_*NORM*_ (*m*,*n*,*i*) will be equal to:

1 $$L_{NORM} \left( {m,n,i} \right) = \frac{{L_{NORM} \left( {m,n,i} \right) - \mathop {\hbox{min} }\limits_{m,n} \left( {L_{MED} \left( {m,n,i} \right)} \right)}}{{\mathop {\hbox{max} }\limits_{m,n} \left( {L_{NORM} \left( {m,n,i} \right) - \mathop {\hbox{min} }\limits_{m,n} \left( {L_{MED} \left( {m,n,i} \right)} \right)} \right)}}$$

The last stage of the initial image processing is removal of uneven illumination. Illumination unevenness is clearly visible almost in every image [20]. In practice in order to remove it, the deduction of the result of low-pass filtration *L*_*GROUND*_ (*m*,*n*,*i*) from the input image, in this case *L*_*NORM*_ (*m*,*n*,*i*), is applied, i.e. the image after removal of unevenness:

2 $$L_{NLOW} \left( {m,n,i} \right) = \left| {L_{NORM} \left( {m,n,i} \right) - L_{GROUND} \left( {m,n,i} \right)} \right|$$

where:

3 $$L_{GROUND} \left( {m,n,i} \right) = L_{NORM} \left( {m,n,i} \right){ \ominus }SE$$

${ \ominus }$ erosion, SE structural element of the size of *M*_*SE*_ × *N*_*SE*_ = *31* × *31* pixels. The output image *L*_*NLOW*_ (*m*,*n*,*i*) is further subject to major image processing.

### Major image processing

Major image processing was performed on the basis of two images: *L*_*GRAY*_ (*m*,*n*,*i*) and *L*_*NLOW*_ (*m*,*n*,*i*). The second one was destined to detect the location of Meibomian glands which are usually arranged vertically (although there may also be other arrangements) in relation to the image plane (positioning of the patient). The analysis is based on using Riesz pyramid i.e. *h*_*R*_ mask defined as:

4 $$h_{R} \,(m_{R} ,n_{R} ,\alpha_{R} ,\sigma_{m} ,\sigma_{n} ,\sigma_{\alpha } ) = \frac{1}{{\sigma_{m} ,\sigma_{n} ,\sigma_{\alpha } \left( {2 \cdot \pi } \right)^{{\frac{3}{2}}} }} \cdot exp\;\left( { - \frac{{m_{R}^{2} }}{{2 \cdot \sigma_{m}^{2} }} - \frac{{n_{R}^{2} }}{{2 \cdot \sigma_{n}^{2} }} - \frac{{\sigma_{R}^{2} }}{{2 \cdot \sigma_{\alpha }^{2} }}} \right)$$

where *σ*_*m*_ *,σ*_*n*_ *,σ*_*α*_ standard deviation of the mean for three dimensions *m*,*n*,*α*, *α* mask rotation angle,* m*_*R*_, *n*_*R*_, *α*_*R*_ normalized values *m, n,α* up to the range from −0.5 to 0.5, i.e. for example for *m*_*R*_:

5 $$m_{R} = \frac{m}{M} - 0.5$$

**Fig. 1:**
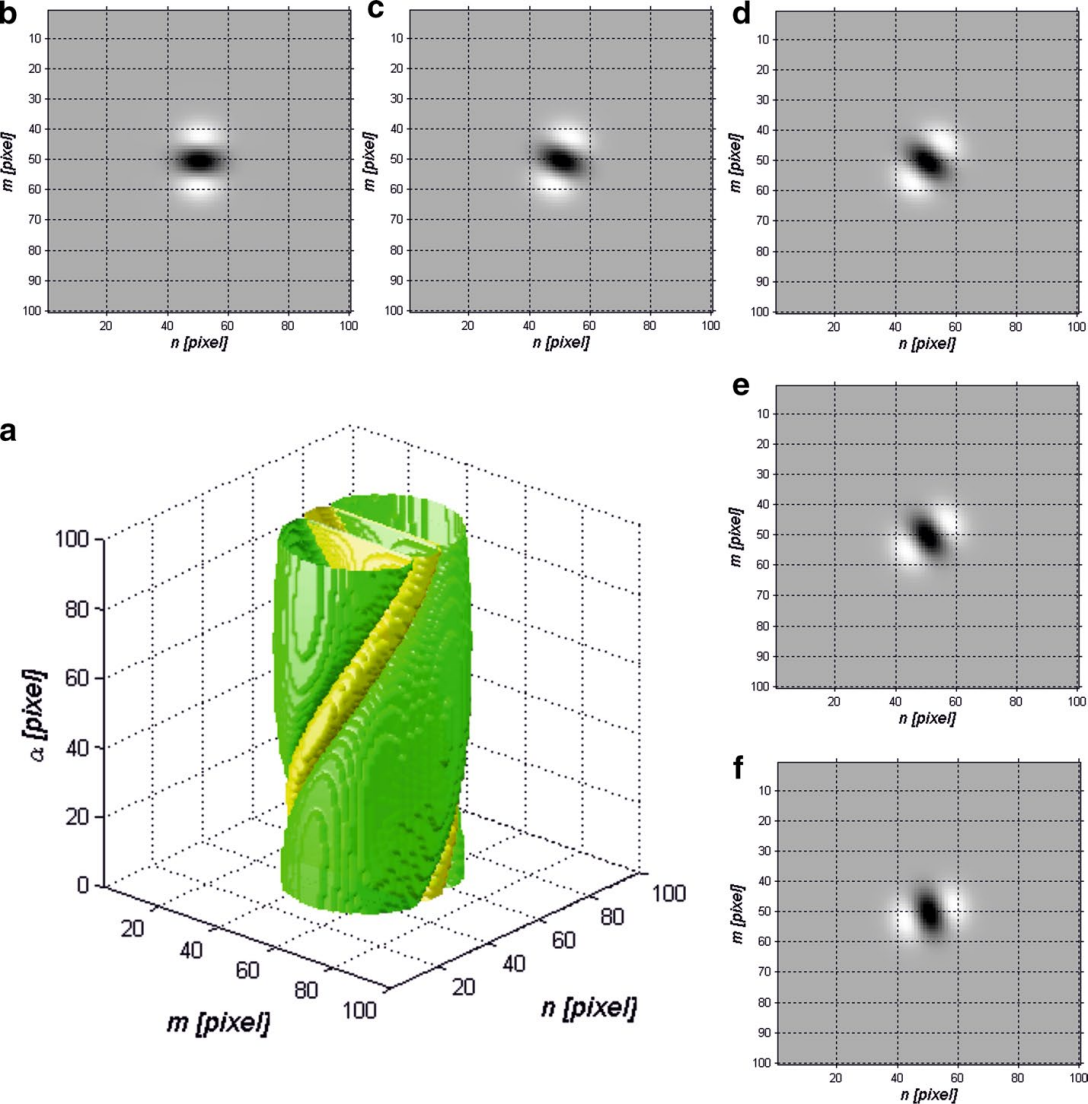
Riesz pyramid, *h*_*R*_ mask defined for *σ*_*m*_ = 0.05*,σ*_*n*_ = 0.05*,σ*_*α*_ = 0.4 and its second derivative calculated after variable *m*: **a** graph of the pyramid of *h*_*R*_ masks sequence in the coordinate system defined for pixel units; **b** *h*_*R*_ mask in the 2D graph for *α* = 0^o^; **c** for *α* = 20^o^; **d** for *α* = 40^o^; **e** for *α* = 60^o^; **f** for *α* = 80^o^

**Fig. 2:**
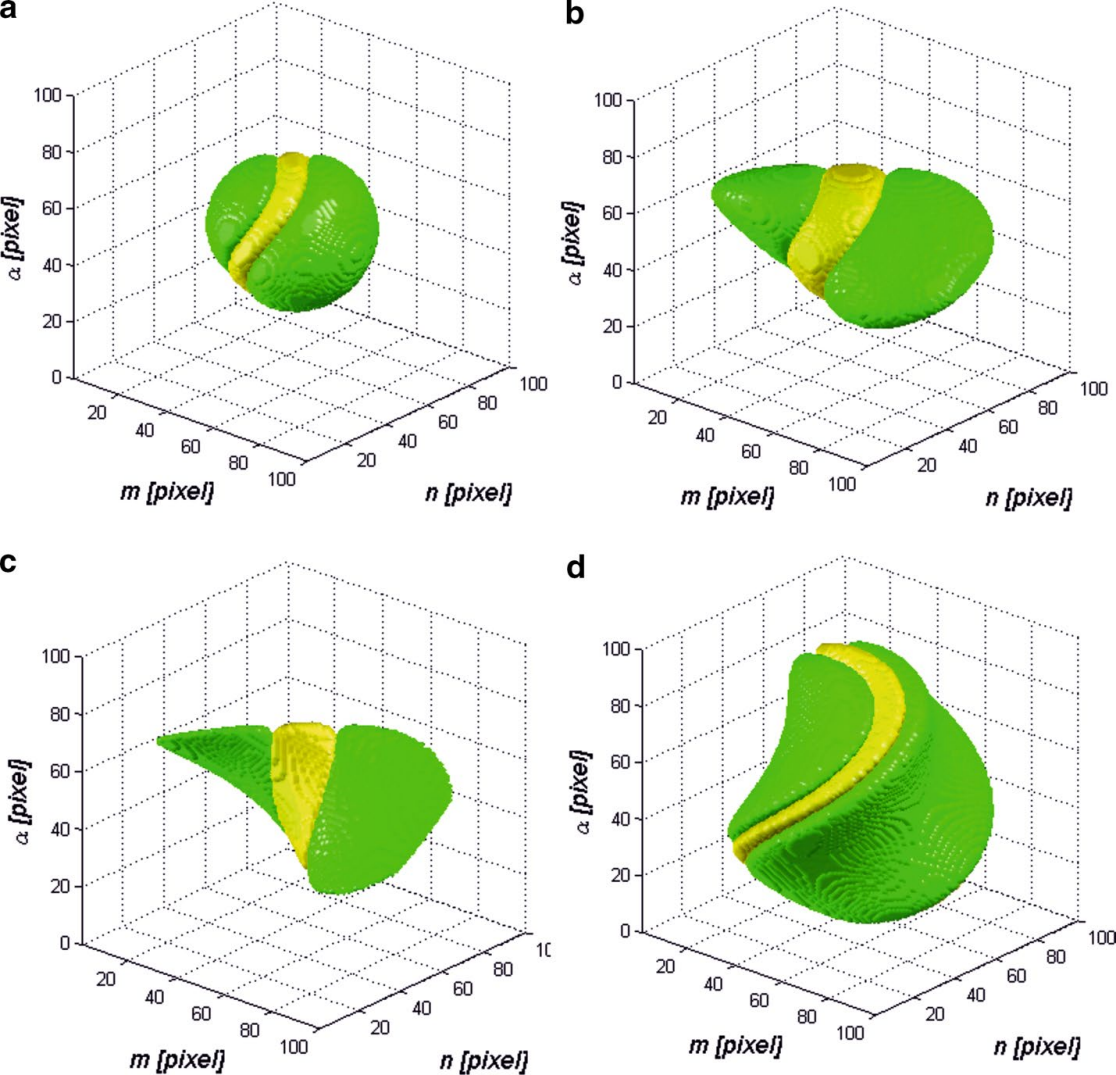
*h*_*R*_ mask defined for **a** *σ*_*m*_ = 0.05*,σ*_*n*_ = 0.05*,σ*_*α*_ = 0.05; **b** *σ*_*m*_ = 0.05*,σ*_*n*_ = 0.1*,σ*_*α*_ = 0.05; **c** *σ*_*m*_ = 0.01*,σ*_*n*_ = 0.1*,σ*_*α*_ = 0.05; **d** *σ*_*m*_ = 0.1*,σ*_*n*_ = 0.05*,σ*_*α*_ = 0.1; and its second derivative calculated after variable *m*

**Fig. 3:**
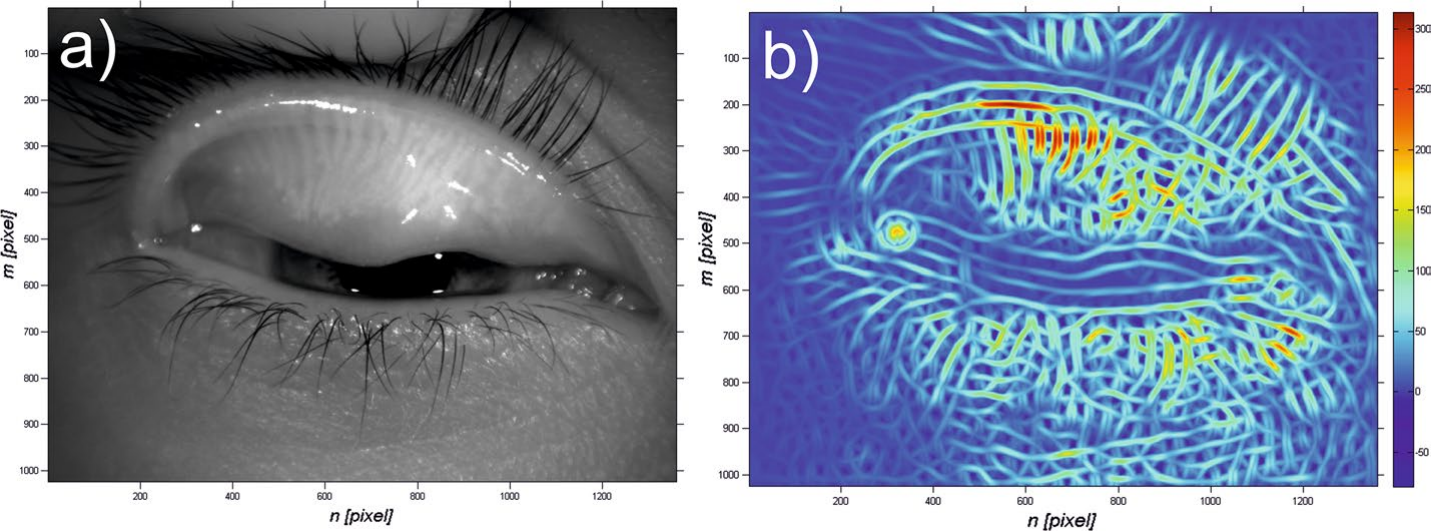
Exemplary input image *L*_*GRAY*_ (*m*,*n*,*i*) **a** and obtained results *L*_*RIESZ*_ (*m*,*n*,*i*) **b** being the convolution for exemplary images *L*_*NLOW*_ (*m*,*n*,*i*) with *h*_*R*_ mask defined for *σ*_*m*_ = 0.05*,σ*_*n*_ = 0.05*,σ*_*α*_ = 0.4 and its second derivative calculated after variable *m*

Riesz pyramid enables creating mask sequences which in turn allow separating Meibomian glands located at various angles relative to image plane and privileged vertical position. In Fig. 1 an exemplary pyramid of *h*_*R*_ mask defined for *h*_*R*_ *σ*_*m*_ = 0.05*,σ*_*n*_ = 0.05*,σ*_*α*_ = 0.4 and second derivative calculated after variable *m* is presented. The obtained results, other forms of *h*_*R*_ mask, are shown in Fig. 2 for other exemplary values *σ*_*m*_ *,σ*_*n*_ and *σ*_*α*_. The obtained results, images *L*_*RIESZ*_ (*m*,*n*,*i*), resulting from the *h*_*R*_ mask convolution for exemplary eye images *L*_*NLOW*_ (*m*,*n*,*i*) are shown in Fig. 3.

The next stage is to designate the area covered by the Meibomian glands. In this case the image *L*_*RIESZ*_ (*m*,*n*,*i*) will be subject to averaging based on the structural element *SE*. This operation will encompass the substitution of areas for which the maximum values of matrix *L*_*RIESZ*_ (*m*,*n*,*i*) have exceeded the set threshold *p*_*r*_ by the value of ‘1’. Therefore, the resulting matrix *L*_*RES*_ (*m*,*n*,*i*) is equal to:

6 $$L_{RES} \left( {m,n,i} \right) = \left\{ {\begin{array}{*{20}l} 1 & {if} & {\mathop {\hbox{max} }\limits_{{m_{S,} n_{S} SE}} \left( {L_{RIESZ} \left( {m + m_{S} ,n + n_{S} ,i} \right)} \right) {\text{>p}}_{\text{r}} } \\ 0 & {other} & \\ \end{array} } \right.$$

where *m*_*S*_ and *n*_*S*_ rows and columns of *SE* mask of the size *M*_*S*_ × *N*_*S*_.

Calculating the maximum value in the area of *SE* mask causes averaging of the information about the location of particular Meibomian glands. Therefore, there occurs globalization of the information concerning the position of Meibomian glands on the lid. However, as a result of this operation there also occurs the covering of the edge of the eyelid, and therefore it must be automatically removed from the resulting analysis. The position of the eyelid edge was calculated as a result of the *xor* operation between two binary images *L*_*RES*_ (*m*,*n*,*i*) and its result of dilation. The binary image of the edge of the eyelid *L*_*BP*_ (*m*,*n*,*i*) therefore equals:

7 $$L_{BP} \left( {m,n,i} \right) = \left( {L_{RES} \left( {m,n,i} \right) \oplus SE2} \right)\underline{ \vee } L_{RES} \left( {m,n,i} \right)$$

where $\oplus$ dilation operator.

The structural element *SE2* of the size of *M*_*S2*_ × *N*_*S2*_ = 60 × 60 pixels with the center of coordinate system placed in the middle of the first row.

**Fig. 4:**
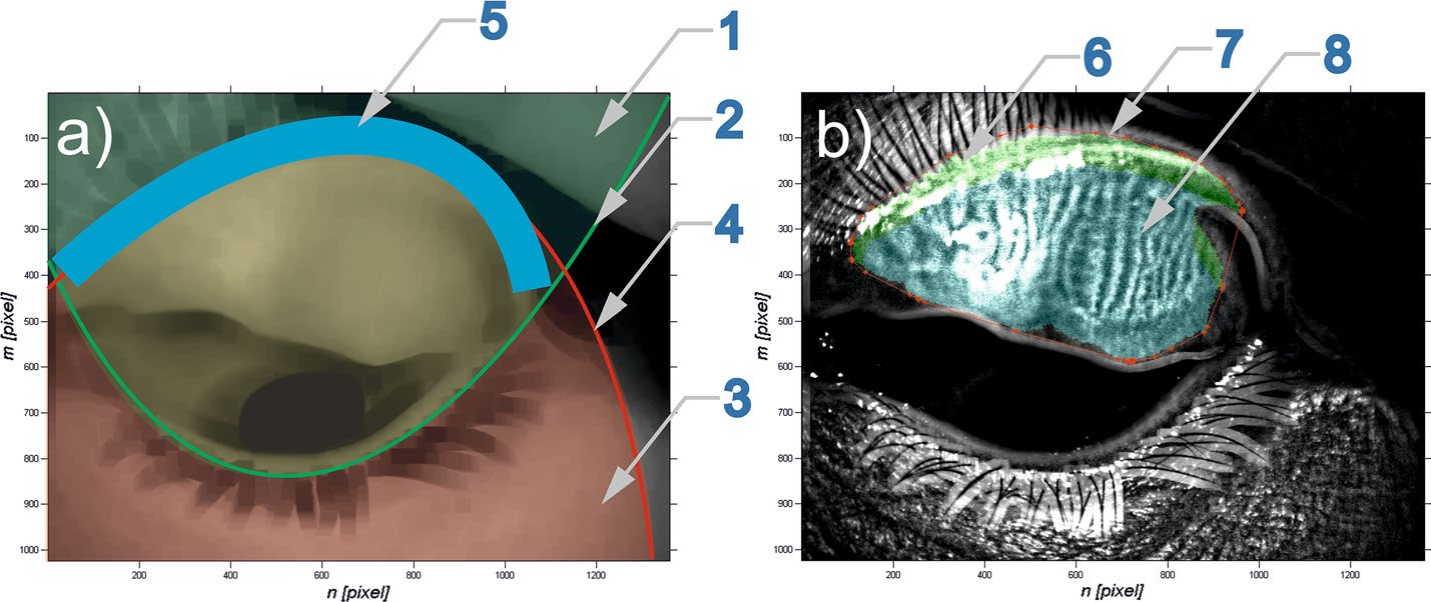
Image analysis results: **a** a demonstrative image showing the location of characteristic, automatically recognized areas: *2* Bézier curve of the lower eyelid boundary and the area *1* marked by it; *4* Bézier curve of the upper eyelid boundary and the area *3* marked by it; *5* area of eyelid edge; **b** image *L*_*NLOW*_ (*m*,*n*,*i*) where *6*- eyelid edge area; *7* the results of manual indication of Meibomian glands area determined by an expert and *8* the area occupied by them calculated automatically

The last operation is to establish the Bézier curve allowing the separation of the area of upper and lower lid—according to Fig. 4. In Fig. 4a a demonstrative image with characteristic automatically recognized areas is presented: 1,2 area and Bézier curve of the lower eyelid boundary; 3,4—are and Bézier curve of upper eyelid boundary; 5—eyelid edge area. Summing up, the new, proposed automatic method of analysis enables automatic calculation of:

- Area occupied by Meibomian glands,
- Area occupied by the eyelid edge,
- Area occupied by the eyelid.

The conducted analysis, particularly the percentage area occupied by Meibomian glands in relation to the whole surface of the eyelid (upper and lower respectively) constitutes the most important result of the proposed new algorithm. In order to determine if the patient is ill or healthy the‚ cut-off’ threshold—*p*_*o*_ was set. Above the *p*_*o*_ threshold the patients are considered to be healthy, while below the threshold they are considered ill. The result will be compared with the analysis conducted manually by specialized doctors from Beijing Institute of Ophthalmology, Beijing Tongren Eye Center, Beijing Tongren Hospital in China. The results of comparison are shown for exemplary patients in Table 1. In Fig. 4b a demonstrative image *L*_*NLOW*_ (*m*,*n*,*i*) was shown with marked digits: 6—area of eyelid edge; 7—the results of manual indication of Meibomian glands area determined by an expert together with the area occupied by them calculated automatically 8. The obtained results in Table 1 concern both healthy and ill patients. According to the expert’s decision they were divided into two groups:

- Group of healthy individuals—Meibomian glands occurring on the whole surface of the eyelid,
- Group of sick individuals—Meibomian glands not occurring on the whole eyelid surface.

**Table 1:** Exemplary obtained results of manual and automatic analysis of Meibomian glands presented in this article

Patient number	Eye—left/right	Eyelid—up/down	Eyelid surface (pixel)	Meibomian glands surface (pixel)	Percentage area of Meibomian glands (%)	Expert’s analysis result (healthy/Ill)
1	Left	Up	59,849	20,131	33	Healthy
2	Left	Down	42,993	13,283	30	Healthy
9	Right	Up	238,269	81,618	34	Healthy
10	Right	Down	48,207	18,449	38	Healthy
14	Right	Down	138,953	28,272	20	Ill
15	Right	Up	206,472	52,585	25	Ill
16	Right	Down	105,846	26,667	25	Ill

**Fig. 5:**
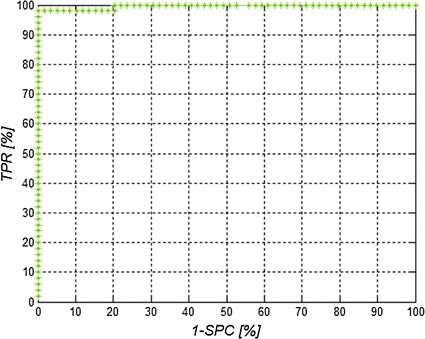
ROC curve for threshold *p*_*o*_ changed in the range of 0–100%

The results of specificity *SPC* and sensitivity *TPR* as well as accuracy defined as *ACC* = *(TN* + *TP)/(TN* + *TP* + *FN* + *FP)* where: *TN*—true negative, *TP*—true positive, *FN*—false negative and *FP*—false positive. The obtained results for 57 patients from 228 images of left and right eye are presented in Table 2. The ROC curve (receiver operating characteristic) as a relationship between sensitivity and specificity for the changed threshold *p*_*o*_ in the range from 0 to 100% is shown in Fig. 5.

**Table 2:** Obtained results of sensitivity, specificity and accuracy for the analyzed images for *p*_*o*_ = 26%

Parameter	Value (%)
*SPC*	100
*TPR*	98
*ACC*	99.08

## Discussion

**Fig. 6:**
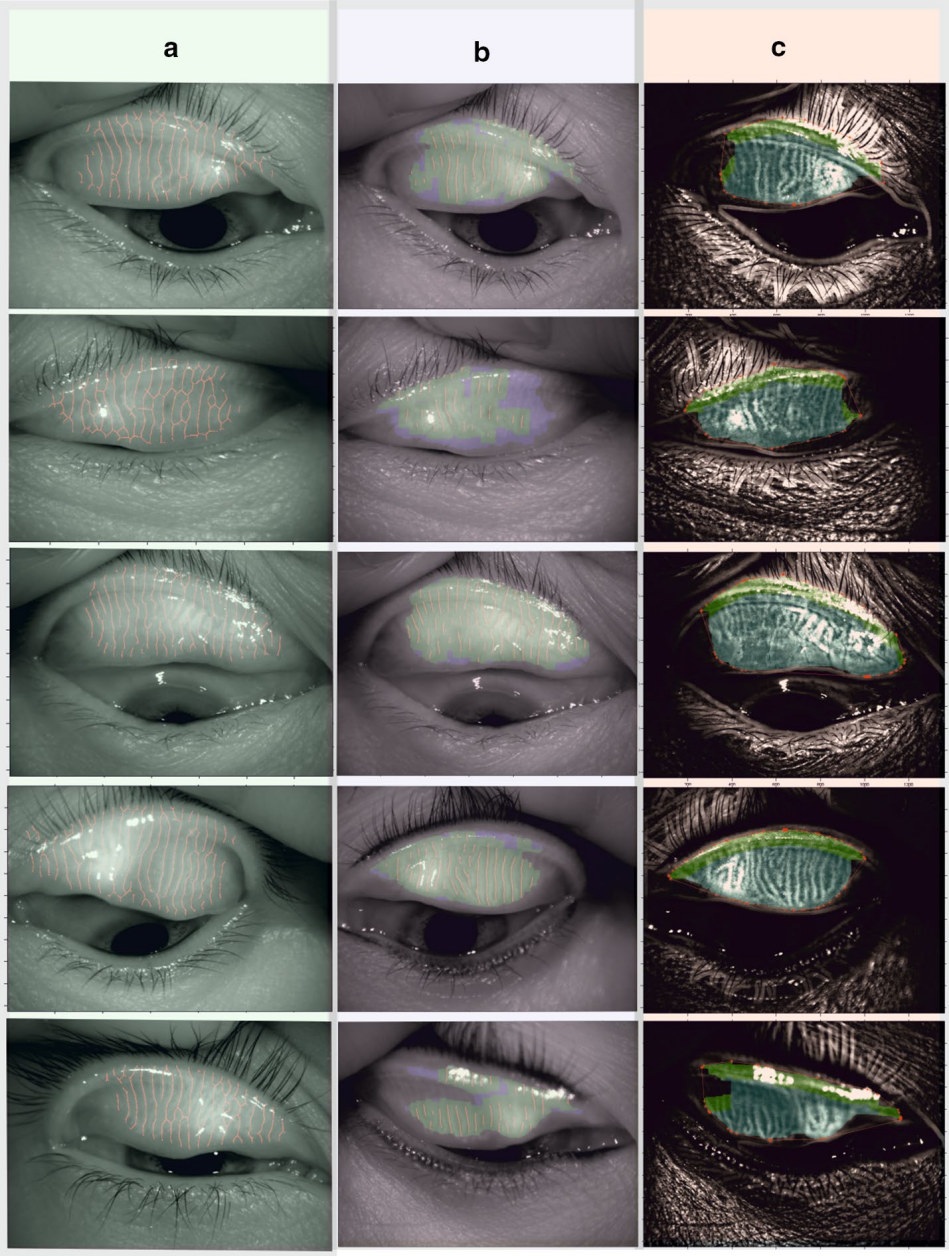
Exemplary results allowing the qualitative comparison of two methods: **a** image with automatically marked location of Meibomian glands according to the method described in [41]; **b** area covering Meibomian glands according to the method described in [41] and **c** results of analysis described in this article (indications complying with indications in **b**)

The developed algorithm was compared with the method proposed in [41]. In the method described in [41] the results of sensitivity were obtained at the level of 99.3% and specificity at 97.5% in the diagnostics of Meibomian glands. The algorithm presented in [41] is not insensitive to the change of parameters, while the speed of image analysis for one patient, according to the Authors’ declaration, does not exceed 0.5 s. This attitude however has disadvantages which involve the necessity of analyzing the location and centerlines of Meibomian glands. The method presented in this article (new algorithm) based on Riesz pyramid of masks is not only faster (average time of analysis for computer with Windows 7 Professional, 64-bit with the Intel Core i7-4960X CPU @ 3.60 GHz is 0.4 s—CPU and memory utilization were the same [41]) but also allows obtaining better sensitivity results equaling 98% and specificity equaling 100%. In Fig. 6 exemplary results comparing two methods are presented: (a) images with automatically marked position of Meibomian glands according to the method described in [41]; (b) area covering Meibomian glands according to the method described in [41] and (c) results of the analysis described in this article (indications complying with indications in Fig. 6b. The key difference visible during comparison of the results is the way of analysis of the areas occupied by Meibomian glands. For the method described in [41] it is also the area occupied by the eyelid edge and therefore included in the area of Meibomian glands. For bigger areas of glands the values are of no significance. However, for small areas occupied by Meibomian glands it is of crucial importance and the resulting consequences are reflected in the values of specificity and sensitivity (97.5 and 99.3% respectively).

## Conclusions

In conclusion, this noncontact meibography system is a useful and patient-friendly method to obtain information about the meibomian gland structure. Using this meibography system, the authors found that changes in Meibomian glands increase with age, develop earlier in men than in women, and are more severe in elderly men than in elderly women. The authors hope that this meibography method will contribute to the elucidation of the pathologic mechanisms of meibomian gland diseases and will help to establish better diagnostic criteria for MGD.

The presented, new method of analysis of Meibomian glands is fully automatic, does not require operator’s intervention, allows obtaining reproducible results and enables a quantitative assessment of Meibomian glands. Compared to the other known methods, particularly with the method described in [41] it allows obtaining better sensitivity and specificity results by 2%. Moreover, it additionally enables removing the area occupied by the eyelid edge. It can be concluded that the analysis of particular Meibomian glands’ location is not an indispensable element and feature. In each case the analysis of percentage area occupied by Meibomin glands in relation to the whole surface of the eyelid is meaningful and adequate.

Currently, the method is being tested at the Beijing Institute of Ophthalmology, Beijing Tongren Hospital in China.

## Abbreviations

MGD: meibomian gland dysfunction
TFLL: tear film lipid layer
DED: dry eye disease
OSDI: ocular surface disease index
BUT: fluorescein tear breakup time
S|T: Schirmer I test

## References

[1] Nicolaides N, Kaitaranta JK, Rawdah TN (1981). Meibomian gland studies: comparison of steer and human lipids. Invest Ophthalmol Vis Sci.

[2] Tomlinson A, Bron AJ, Korb DR (2011). The international workshop on meibomian gland dysfunction: report of the diagnosis subcommittee. Invest Ophthalmol Vis Sci.

[3] Schein OD, Munoz B, Tielsch JM (1997). Prevalence of dry eye among the elderly. Am J Ophthalmol.

[4] Lin PY, Tsai SY, Cheng CY (2003). Prevalence of dry eye among an elderly Chinese population in Taiwan: the Shihpai Eye Study. Ophthalmology.

[5] Jie Y, Xu L, Wu YY (2009). Prevalence of dry eye among adult Chinese in the Beijing Eye Study. Eye (Lond).

[6] Siak JJ, Tong L, Wong WL (2012). Prevalence and risk factors of meibomian gland dysfunction: the Singapore Malay eye study. Cornea.

[7] Bron AJ, Tiffany JM, Gouveia SM (2004). Functional aspects of the tear film lipid layer. Exp Eye Res.

[8] Nichols KK, Foulks GN, Bron AJ (2011). The international workshop on meibomian gland dysfunction: executive summary. Invest Ophthalmol Vis Sci.

[9] Baudouin C, Messmer EM, Aragona P (2016). Revisiting the vicious circle of dry eye disease: a focus on the pathophysiology of meibomian gland dysfunction. Br J Ophthalmol.

[10] Nichols KK, Foulks GN, Bron AJ (2011). The international workshop on meibomian gland dysfunction: executive summary. Invest Ophthalmol Vis Sci.

[11] Tomlinson A, Bron AJ, Korb DR (2011). The international workshop on meibomian gland dysfunction: report of the diagnosis subcommittee. Invest Ophthalmol Vis Sci.

[12] Geerling G, Tauber J, Baudouin C (2011). The international workshop on meibomian gland dysfunction: report of the subcommittee on management and treatment of meibomian gland dysfunction. Invest Ophthalmol Vis Sci.

[13] Lemp MA, Crews LA, Bron AJ (2012). Distribution of aqueous-deficient and evaporative dry eye in a clinic-based patient cohort: a retrospective study. Cornea.

[14] Cho P, Yap M (1993). Schirmer test. I. A review. Optom Vis Sci.

[15] Doughty MJ (2014). Fluorescein-tear breakup time as an assessment of efficacy of tear replacement therapy in dryeye patients: a systematic review and meta-analysis. Ocular Surface.

[16] Alghamdi YA, Mercado C, McClellan AL, Batawi H, Karp CL, Galor A (2016). Epidemiology of meibomian gland dysfunction in an elderly population. Cornea.

[17] Chhadva P, McClellan AL, Alabiad CR, Feuer WJ, Batawi H, Galor A (2016). Impact of eyelid laxity on symptoms and signs of dry eye disease. Cornea.

[18] Ablamowicz AF, Nichols JJ, Nichols KK (2016). Association between serum levels of testosterone and estradiol with meibomian gland assessments in postmenopausal women. Invest Ophthalmol Vis Sci.

[19] Jester JV, Parfitt GJ, Brown DJ (2015). Meibomian gland dysfunction: hyperkeratinization or atrophy?. BMC Ophthalmol..

[20] Koprowski R, Wilczyński S, Wróbel Z, Kasperczyk S, Błońska-Fajfrowska B (2014). Automatic method for the dermatological diagnosis of selected hand skin features in hyperspectral imaging. Biomed Eng Online..

[21] La Porta Weber S, Becco de Souza R, Gomes JÁ, Hofling-Lima AL (2016). The use of the esclera scleral contact lens in the treatment of moderate to severe dry eye disease. Am J Ophthalmol.

[22] Cox SM, Nichols JJ (2015). Association between meibomian gland testing and ocular surface sensitivity. Cornea.

[23] Moy A, McNamara NA, Lin MC (2015). Effects of isotretinoin on Meibomian glands. Optom Vis Sci..

[24] Ezuddin NS, Alawa KA, Galor A (2015). Therapeutic strategies to treat dry eye in an aging population. Drugs Aging..

[25] Tong L, Chaurasia SS, Mehta JS, Beuerman RW (2010). Screening for Meibomian gland disease: its relation to dry eye subtypes and symptoms in a tertiary referral clinic in Singapore. Invest Ophthalmol Vis Sci.

[26] Kamao T, Yamaguchi M, Kawasaki S, Mizoue S, Shiraishi A, Ohashi T (2011). Screening dry eye with newly developed ocular surface thermographer. Am J Ophthalmol.

[27] Su TY, Hwa CK, Liu PH (2011). Noncontact detection of dry eye using a custom designed infrared thermal image system. J Biomed Opt.

[28] Pult H, Riede-Pult BH, Nichols JJ (2012). Relation between upper and lower lids’ Meibomain gland morpohology tear film and dry eye. Opt Vis Sci.

[29] Pult H, Nichols JJ (2012). A review of Meibography. Opt Vis Sci.

[30] Pult H, Riede-Pult BH. An assessment of subjective and objective grading of Meibography images; ARVO 2012.

[31] Arita R, Itoh K, Inoue K, Amano S (2008). Noncontact infrared Meibography to document age-related changes of the Meibomian glands in a normal population. Ophthalmology.

[32] Arita R, Itoh K, Maeda S (2009). Proposed diagnostic criteria for obstructive meibomian gland dysfunction. Ophthalmology.

[33] Srinivasan S, Menzies K, Sorbara L, Jones L (2012). Infrared imaging of meibomian gland structure using a novel keratograph. Opt Vis Sci.

[34] Głowacz A, Głowacz Z (2016). Diagnostics of stator faults of the single-phase induction motor using thermal images. MoASoS and selected classifiers. Measurement.

[35] Głowacz A, Głowacz Z (2016). Recognition of images of finger skin with application of histogram, image filtration and K-NN classifier. Biocybern Biomed Eng.

[36] Ji Chunhong, Jinhua Yu, Li Tianjie, Tian Lei, Huang Yifei, Wang Yuanyuan, Zheng Yongping (2015). Dynamic curvature topography for evaluating the anterior corneal surface change with Corvis ST. BioMed Eng Online.

[37] Porwik P, Orczyk T, Lewandowski M, Cholewa M (2016). Feature projection *k*-NN classifier model for imbalanced and incomplete medical data. Biocybernet Biomed Eng..

[38] Koprowski R (2014). Automatic method of analysis and measurement of additional parameters of corneal deformation in the Corvis tonometer. Biomed Eng Online..

[39] Celika T, Leea HK, Petznickb A, Tongb L (2013). Bioimage informatics approach to automated meibomian gland analysis in infrared images of meibography. J Optom..

[40] Koprowski R, Wilczynski S, Nowinska A (2015). Quantitative assessment of responses of the eyeball based on data from the Corvis tonometer. Comput Biol Med.

[41] Koprowski R, Wilczyński S, Olczyk P, Nowińska A, Węglarz B, Wylęgała E (2016). A quantitative method for assessing the quality of Meibomian glands. Comput Biol Med.

